# Asymmetrical high-flow nasal cannula performs similarly to standard interface in patients with acute hypoxemic post-extubation respiratory failure: a pilot study

**DOI:** 10.1186/s12890-023-02820-x

**Published:** 2024-01-08

**Authors:** Annalisa Boscolo, Tommaso Pettenuzzo, Francesco Zarantonello, Nicolò Sella, Elisa Pistollato, Alessandro De Cassai, Sabrina Congedi, Irene Paiusco, Giacomo Bertoldo, Silvia Crociani, Francesca Toma, Giulia Mormando, Giulia Lorenzoni, Dario Gregori, Paolo Navalesi

**Affiliations:** 1 Department of Medicine (DIMED), University of Padua, Padua, Italy; 2 Institute of Anaesthesia and Intensive Care, Padua University Hospital, 13, Giustiniani Street, Padua, 35128 Italy; 3 Thoracic Surgery and Lung Transplant Unit, Department of Cardiac, Thoracic, Vascular Sciences, and Public Health, University of Padua, Padua, Italy; 4 Emergency Department, Padua University Hospital, Padua, Italy; 5 Unit of Biostatistics, Epidemiology, and Public Health, Department of Cardiac, Vascular Sciences, and Public Health, University of Padua, Thoracic, Padua, Italy

**Keywords:** High-flow nasal cannula, High flow nasal oxygen, High flow nasal therapy, Asymmetrical cannula, Asymmetrical cannula, DUET

## Abstract

**Background:**

Standard high-flow nasal cannula (HFNC) is a respiratory support device widely used to manage post-extubation hypoxemic acute respiratory failure (hARF) due to greater comfort, oxygenation, alveolar recruitment, humidification, and reduction of dead space, as compared to conventional oxygen therapy. On the contrary, the effects of the new asymmetrical HFNC interface (Optiflow® Duet system (Fisher & Paykel, Healthcare, Auckland, New Zealand) is still under discussion. Our aim is investigating whether the use of asymmetrical HFNC interface presents any relevant difference, compared with the standard configuration, on lung aeration (as assessed by end-expiratory lung impedance (EELI) measured by electrical impedance tomography (EIT)), diaphragm ultrasound thickening fraction (TFdi) and excursion (DE), ventilatory efficiency (estimated by corrected minute ventilation (MV)), gas exchange, dyspnea, and comfort.

**Methods:**

Pilot physiological crossover randomized controlled study enrolling 20 adults admitted to the Intensive Care unit, invasively ventilated for at least 24 h, and developing post-extubation hARF, i.e., PaO_2_/set FiO_2_ < 300 mmHg during Venturi mask (VM) within 120 min after extubation. Each HFNC configuration was applied in a randomized 60 min sequence at a flow rate of 60 L/min.

**Results:**

Global EELI, TFdi, DE, ventilatory efficiency, gas exchange and dyspnea were not significantly different, while comfort was greater during asymmetrical HFNC support, as compared to standard interface (10 [7–10] and 8 [7–9], p-value 0.044).

**Conclusions:**

In post-extubation hARF, the use of the asymmetrical HFNC, as compared to standard HFNC interface, slightly improved patient comfort without affecting lung aeration, diaphragm activity, ventilatory efficiency, dyspnea and gas exchange.

**Clinical trial number:**

ClinicalTrial.gov. Registration number: NCT05838326 (01/05/2023).

**New & noteworthy:**

The asymmetrical high-flow nasal cannula oxygen therapy (Optiflow® Duet system (Fisher & Paykel, Healthcare, Auckland, New Zealand) provides greater comfort as compared to standard interface; while their performance in term of lung aeration, diaphragm activity, ventilatory efficiency, dyspnea, and gas exchange is similar.

**Supplementary Information:**

The online version contains supplementary material available at 10.1186/s12890-023-02820-x.

## Introduction

In recent years, high-flow nasal cannula (HFNC) oxygen therapy has become popular among intensivists to manage patients with hypoxemic acute respiratory failure (hARF) [1]. According to the last European Respiratory Society task force, HFNC is a valuable intervention for improving lung aeration, oxygenation and alveolar recruitment in different populations, such as post-operative patients and nonsurgical subjects at risk of extubation failure or pulmonary complications [1–3].

HFNC delivers up to 60 L/min of warmed humidified gas, with an inspired fraction of oxygen (FiO_2_) ranging from 21 to 100% [4, 5]. HFNC promotes naso-pharyngeal dead space washout, leading to a decrease of minute ventilation and diaphragm activity, and may increase to some extent the end-expiratory lung volume (EELV) consequent to a variable rise of end-expiratory airway pressure [2, 5, 6]. Finally, by delivering warm and well humidified gas, HFNC may facilitate the clearance of tracheobronchial secretions. Overall, HFNC has the potential to improve oxygenation and patient comfort, while increasing EELV and reducing inspiratory effort [5, 7, 8].

Indeed, they have been tested as first-line treatment for avoiding intubation in patients experiencing hARF or exacerbations of chronic obstructive pulmonary disease (COPD), for preventing re-intubation, especially in nonsurgical patients at low or moderate risk of extubation failure or in post-operative patients at low or high risk of pulmonary complications, and for preoxygenation during endotracheal intubation [5, 7–16].

Recently, a new HFNC interface using asymmetrical prongs was approved for clinical practice [17, 18]. Unlike standard nasal cannulas with equally sized prongs, the asymmetrical prongs deliver different flow rates between the two nostrils [17, 18]. Bench studies have demonstrated that the asymmetrical configuration resulted in higher positive end-expiratory pressure (PEEP) and accelerated clearance of the anatomical dead space [2, 17]. As compared to the conventional HFNC interface, Slobod and colleagues recently found the asymmetrical interface to be associated only with reduced minute ventilation and work of breathing in 10 non-intubated patients with mild-to-moderate hARF, likely attributable to the enhanced carbon dioxide (CO_2_) clearance from the upper airway [18].

However, no study has, in so far, focused on patients developing hARF after extubation and on assessing the effect of the asymmetrical HFNC interface to prevent extubation failure.

We designed this pilot study for investigating whether in patients developing hARF early after extubation, the use of asymmetrical HFNC interface presents any relevant difference, compared with the standard configuration, on lung aeration - as assessed by end-expiratory lung impedance (EELI) measured by electrical impedance tomography (EIT) -, on diaphragm ultrasound thickening fraction (TFdi) and excursion (DE) -, ventilatory efficiency - estimated by corrected minute ventilation (MV) -, gas exchange, dyspnea, and comfort.

## Materials and methods

This pilot physiological crossover randomized controlled study included all consecutive adult patients, admitted to the intensive care unit (ICU) of the University Hospital of Padua (Italy) between May 8th and June 10th 2023, undergoing invasive mechanical ventilation for at least 24 h and experiencing post-extubation hARF, defined as arterial partial pressure of oxygen (PaO_2_) to set inspiratory fraction of oxygen (FiO_2_) ratio < 300 mmHg during VenturiMask (VM) support [8], within 120 min after extubation. Exclusion criteria were: (i) long-term oxygen therapy, (ii) need for rescue noninvasive ventilation after extubation (based on predefined criteria [19]), (iii) chronic pulmonary disease, (iv) moderate-severe cardiac failure, (v) severe acute respiratory syndrome coronavirus-2 infection, (vi) pregnancy, (vii) presence of tracheostomy, (viii) contraindications to EIT [20] or HFNC interface [4, 21], and *(ix)* requiring nasogastric tubes for mandatory clinical reasons, i.e., delayed gastric emptying, upper abdominal surgery.

Attending ICU physicians identified patients as ready to undergo the first spontaneous breathing trial when they met the following predefined criteria in a daily screening, as previously described [22–24]: (1) PaO_2_/FiO_2_ ≥ 200 mmHg with PEEP ≤ 8 cmH_2_O and FiO_2_ ≤ 0.4; (2) respiratory rate (RR) ≤ 30/min (during pressure support ventilation); (3) a cooperative cognitive state (Richmond agitation-sedation scale between 0 and − 1); and (4) hemodynamic stability (heart rate < 140 beats min^−1^ and mean arterial pressure > 60 mmHg with norepinephrine < 0.1 mcg/kg/min or dobutamine < 5 mcg/kg/min and without epinephrine). After a 30-minute spontaneous breathing trial, the patient was extubated only in the absence of any of these criteria: (1) signs of acute respiratory distress (RR ≥ 35/min, agitation, recruitment of accessory muscles, and peripheral oxygen saturation < 90%); (2) life-threatening cardiac arrhythmias; (3) copious secretions [22–24].

The study was approved by the Institutional Ethics Committee of Padua University Hospital (reference number: AOP2949) and registered on ClinicalTrial.gov (registration number NCT05838326, 01/05/2023). The study was carried out according to the Declaration of Helsinki and written informed consent was obtained from all patients. The asymmetrical HFNC interfaces were kindly provided by Fisher & Paykel Healthcare (New Zealand) only for research purposes, without any economic interests. The industry was not involved in any phase of the study.

### Randomization

Randomization was performed within 120 min after extubation, immediately after validation of the oxygenation criteria, defined as PaO_2_/set FiO_2_ < 300 mmHg during VM support. According to a web-based blocked random sequence, all patients received HFNC therapy through the asymmetrical interface Optiflow® Duet system (Fisher & Paykel, Healthcare, Auckland, New Zealand) and through the conventional interface.

Either with conventional HFNC or asymmetrical device, the set FiO_2_ was titrated to maintain a peripheral oxygen saturation between 92% and 98%, the gas flow rate was set at 50–60 L/min (AIRVO 2, Fisher&Paykel Healthcare, New Zealand), based on the size of the nostril, and the temperature of the heated humidifier (Fisher&Paykel Healthcare, New Zealand) was set at 37° C (absolute humidity delivered 44 mgH_2_O/L) for the entire study period. Each step was 60 min long and a 10-min ‘wash-out’ phase with VM support was required before each step. The standard and asymmetrical interfaces were identically sized, i.e., small, medium, or large, according to the distance between the patient’s nostrils, as recommended by the manufacturers [21].

Baseline demographic and clinical data were collected from electronic health records. During the last 10 min of each phase (i.e., MV, standard HFNC, and asymmetrical HFNC) the following variables were collected: respiratory rate, pH, arterial oxygen saturation (SaO_2_), PaO_2_/set FiO_2_, arterial pressure of carbon dioxide (PaCO_2_) and FiO_2_, systolic blood pressure, diastolic blood pressure, comfort, dyspnea, and EIT and ultrasound variables. Comfort and dyspnea were evaluated using a numerical scale (NRS) (ranging between 0 and 10) and the Borg scale, respectively [7, 25]. All patients were blinded to the novelty of the asymmetrical interface.

### EIT

After meeting the inclusion criteria, a 16-electrode EIT belt was placed around the chest, as previously described [20, 26]. The following EIT parameters were recorded during the last 10 min of each step and before ultrasound assessment: *(i)* the average global tidal volume (V_T_) and the percentage of V_T_ distributed to non-dependent and dependent lung regions (V_T_glob, V_T_non-dep, and V_T_dep, respectively); *(ii)* the MV and the corrected MV, calculated as [(V_T_glob x PaCO_2_)/40 mmHg]* respiratory rate per minute^− 1^, where 40 mmHg is the ideal value of PaCO_2_ [27, 28]; *(iii)* the global and regional changes in EELI (estimating EELV) during the VM and in each HFNC phase (ΔEELIglob, ΔEELInon-dep, and ΔEELIdep, respectively); *(iv)* the global inhomogeneity index (GI) and the regional ventilation delay (RVD) [29, 30].

### Diaphragm ultrasound

Diaphragm ultrasound evaluation was performed at the bedside, during quiet breathing, with the patient in a semi-recumbent position, by two trained intensivists (AB and TP) [19], using a 4–12 MHz linear array transducer (Mindray M9, North America, NJ, USA), placed perpendicular to the right chest wall between the 9th and 10th intercostal spaces (at the level of apposition) after the EIT evaluation, as previously published [19, 31].

The diaphragm thickness was measured at both end-expiration and inspiration, and TFdi was calculated as the average of three respiratory cycles, according to the formula: TFdi (%) = (inspiratory thickness-expiratory thickness)/expiratory thickness*100 [32]. Diaphragm ultrasound assessment was performed only on the right side due to the lower interobserver reproducibility on the left side [19, 33, 34]. The intra- and inter-observer agreement between the two observers was previously published [19].

DE was measured with a low frequency curved array probe (2–5 MHz) positioned just below the costal arch at the midclavicular line and by angling the ultrasound beam as much as possible cranially and perpendicular to the diaphragmatic dome. The diaphragm is identified as a bright line that covers the liver and the spleen. During inspiration, the diaphragm should move toward the probe [32, 35]. Excursion is quantified in M-mode, with the M-line placed perpendicularly to the direction of motion, and as the mean of three quiet breathings [32, 36].

### Statistical analysis

Continuous data are presented as median and interquartile range [IQR]. Being a pilot study, a sample size was not calculated a priori. Comparisons between different interfaces were analyzed using the Wilcoxon signed rank test for paired data and all p-values were adjusted by Benjamini and Hochberg method. Missing data was omitted from the analysis. Subset analyses were performed according to the improvement on lung aeration during the asymmetrical support. All statistical tests were two-tailed and statistical significance was defined by p < 0.05. Analyses were conducted using Prism (version 5.0; GraphPad Software, Inc, La Jolla, CA, USA) and R (version 4.0.3, R Foundation for Statistical Computing, Vienna, Austria).

## Results

**Fig. 1:**
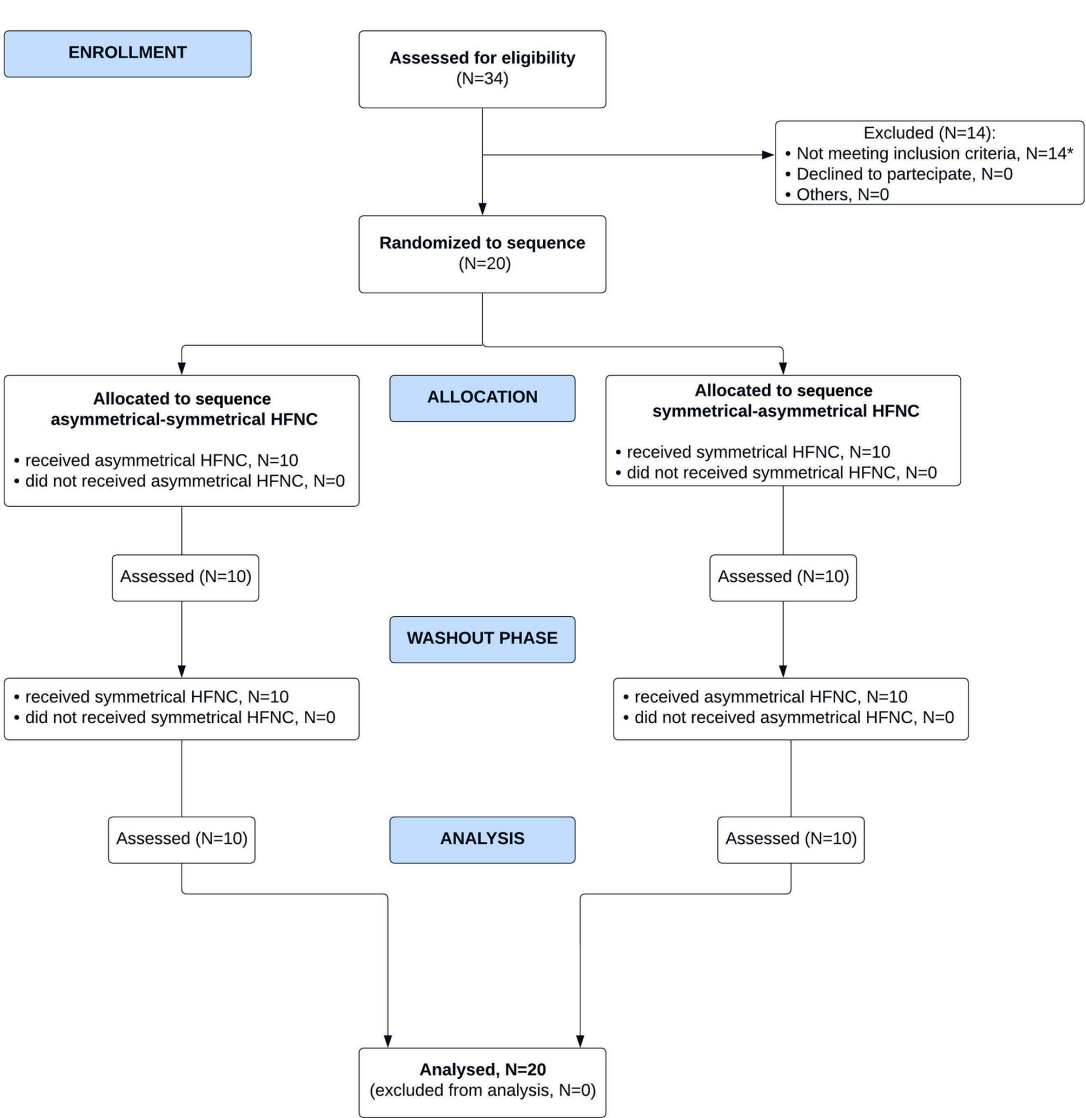
CONSORT flow diagram for crossover trials. ^*^Patients not meeting inclusion criteria: rescue noninvasive ventilation after extubation N = 5, chronic pulmonary disease N = 2, tracheostomy N = 2, mandatory nasogastric tube N = 5. *Abbreviations*: *HFNC* high flow nasal cannula, *N* number, *EIT* electrical impedance tomography

As shown in Fig. 1, we evaluated for enrollment 35 patients and excluded 14 patients not meeting inclusion criteria. One patient was dropped out because of inadequate EIT recordings, leaving 20 patients eligible for analysis.

Patients’ characteristics are reported in Table 1. Median age was 65 [55–76] years and seven (35%) were female. The most frequent etiology for admission to the ICU was elective abdominal surgery (35%), followed by septic shock (15%) and trauma (15%) (Table 1). Extubation occurred after the first spontaneous breathing trial in 17 out of 20 (85%) patients, and after the second attempt in 3 (15%) patients. During VM support, PaO_2_/FiO_2_ was 195 [177–259] mmHg. The total duration of invasive mechanical ventilation was 30 [27–84] hours, and ICU stay was 2 [2–5] days. One patient (5%) died during the ICU stay (Table 1).

**Table 1:** Characteristics of the study population

N	Gender	Size cannula	Age (years)	BMI (kg/m^2^)	SAPS II	ICU admission	IMV (hours)	ICU LOS (days)	PaO_2_/FiO_2_ (Venturi mask)
1	F	S	52	27	21	Trauma	84	7	173
2	M	L	84	25	52	Septic shock	131	5	263
3	F	M	77	26	47	Septic shock	281	14	169
4	M	L	54	29	49	Septic shock	320	14	145
5	F	M	53	21	34	Abdominal surgery	30	2	283
6	M	M	60	27	23	Neurosurgery	30	3	176
7	M	L	73	26	40	Abdominal surgery	25	2	279
8	F	M	77	27	40	Gastrointestinal bleeding	40	14	213
9	F	M	73	21	48	Abdominal surgery	28	2	184
10	M	L	65	26	38	Trauma	27	2	194
11	M	L	59	27	18	Endocrinological surgery	27	2	219
12	M	L	85	28	34	Neurosurgery	28	2	186
13	F	S	69	22	34	Abdominal surgery	28	2	245
14	M	L	68	27	38	Abdominal surgery	28	2	196
15	M	M	33	41	40	Trauma	29	2	183
16	M	M	52	28	26	Gastrointestinal bleeding	288	4	225
17	M	M	79	27	40	Thoracic surgery	83	4	180
18	F	M	64	26	29	Abdominal surgery	25	2	290
19	M	L	63	29	45	Otorhinolaryngological surgery	36	3	163
20	M	M	60	33	18	Abdominal surgery	25	2	272
Total 20	F (35%)	-	65 [55–76]	27 [26–29]	38 [27–44]	-	30 [27–84]	2 [2–5]	195 [177–259]

Continuous variables are expressed as median, with interquartile range [IQR], and categorical variables are expressed as absolute values (%)

*Abbreviations*: *N* patient number, *Tot* Total, *BMI* body mass index, *SAPS II* simplified acute physiology score II at ICU admission, *ICU* intensive care unit, *IMV* invasive mechanical ventilation, *LOS* length of stay, *PaO*_*2*_ partial arterial pressure of oxygen, *FiO*_*2*_ inspiratory fraction of oxygen

### Lung aeration and diaphragm activity

**Fig. 2:**
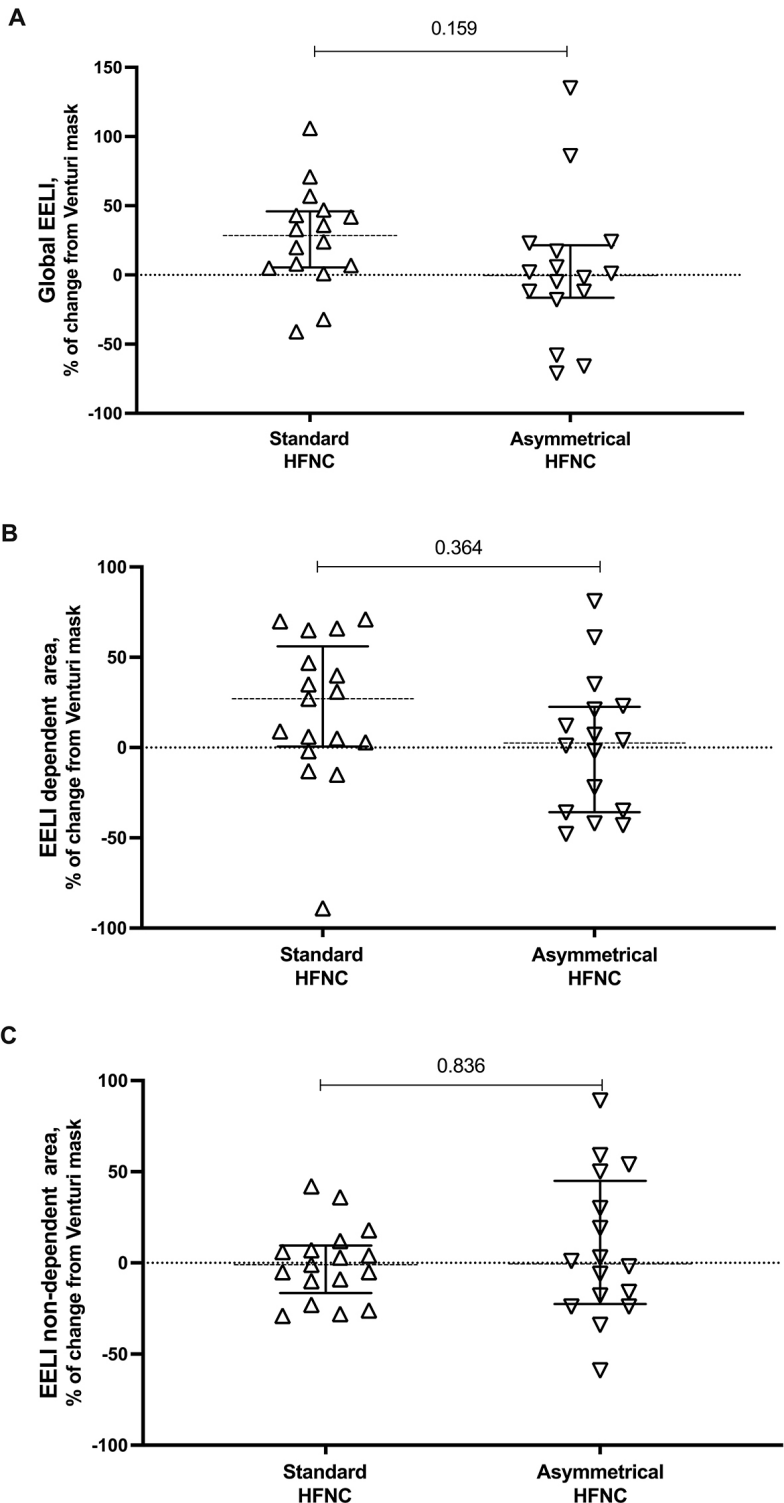
Electrical impedance tomography during standard and asymmetrical HFNC oxygen therapy. Variables are expressed as median, with an interquartile range [IQR]. Additional data are reported in Fig. S1. **A**: global lung aeration; **B**: lung aeration in dependent area; **C**: lung aeration in non-dependent area. *Abbreviations*: *ns* not significant, *HFNC* high-flow nasal cannula, *dep* dependent, *non-dep* non-dependent, *EELI* end-expiratory lung impedance (measured as percent change from VM)

**Fig. 3:**
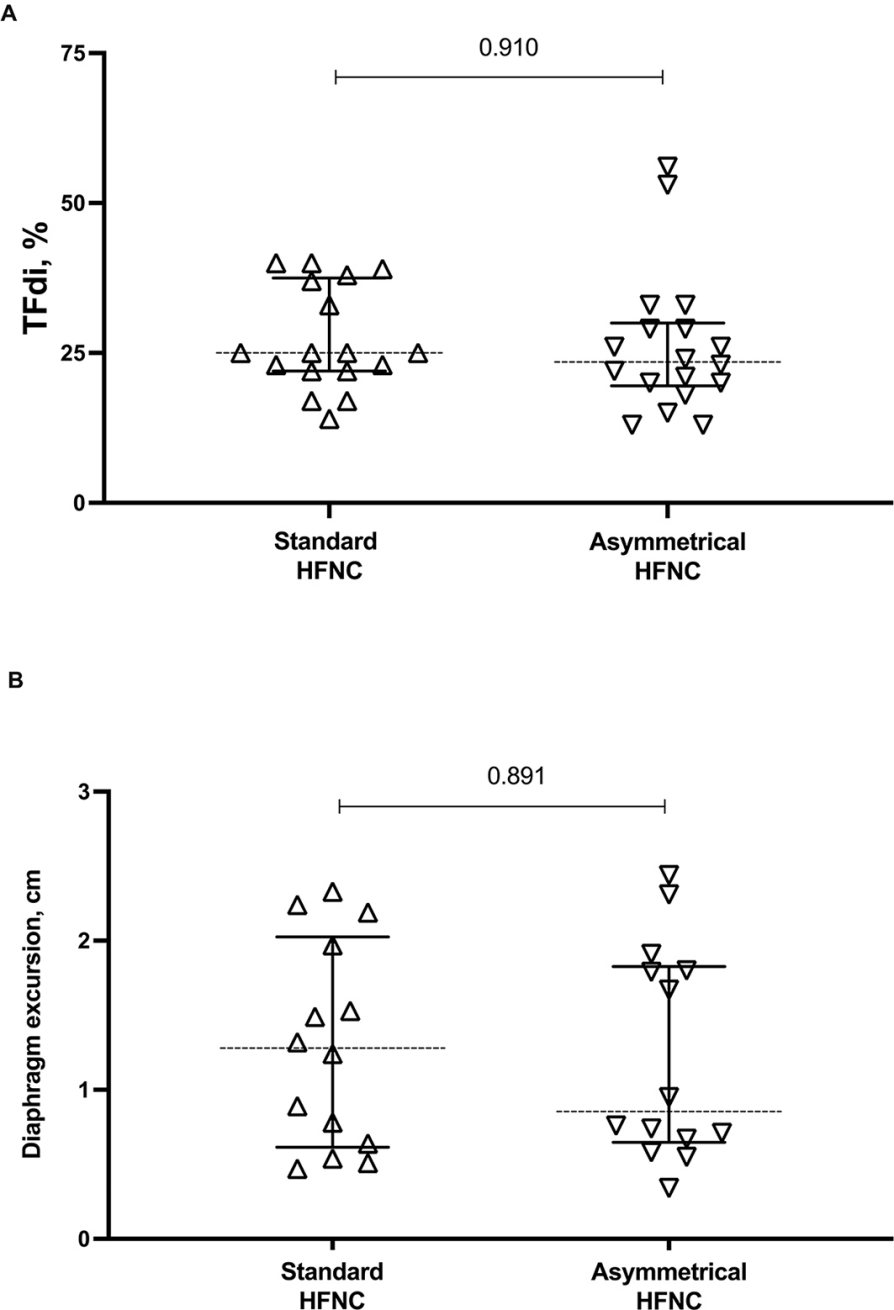
Diaphragm ultrasound evaluation during standard and asymmetrical HFNC oxygen therapy. Variables are expressed as median, with an interquartile range [IQR]. Additional data are reported in Fig. S2. **A**: TFdi; **B**: diaphragm excursion. *Abbreviations*: *HFNC* high-flow nasal cannula, *TFdi* diaphragm thickening fraction

As shown in Fig. 2 and S1, no differences were found in the percent change of global EELI (p = 0.159) and its distribution in dependent (p = 0.364) and not-dependent (p = 0.836) lung regions when passing from VM and asymmetrical or standard HFNC. Also, MV and corrected MV were similar between asymmetrical and standard HFNC, as well as global V_T_, GI and RVD (Table 2). Furthermore, TFdi (p = 0.910) and DE (p = 0.891) were not different in the two HFNC phases (Fig. 3 and S2).

**Table 2:** Electrical impedance tomography parameters

Variable	Standard HFNC^a^ (N = 20)	Asymmetrical HFNC^b^ (N = 20)	Adjusted p-value (a-b)	Venturi mask^c^ (n = 20)	Adjusted p-value (b-c)
MV (change from VM), %	−13 [−26, 1]	−12 [−18, −2]	0.731	–	0.145*
Corrected MV (change from VM), %	−10 [−21, 3]	−11 [−19, −0.10]	0.992	–	0.050*
Global V_T_, (change from VM), %	106 [87–130]	100 [94–113]	0.654	–	0.749
V_T non−dep_, %	57 [31–66]	58 [41–66]	0.731	53 [42–60]	0.749
V_T dep_, %	51 [37–68]	51 [39–57]	0.731	48 [41–59]	0.936
Ratio V_T non−dep/dep_	1.04 [0.60–1.49]	1.12 [0.71–1.53]	0.843	1.10 [0.80–1.50]	0.749
GI index	48 [38–51]	49 [45–65]	0.654	49 [42–73]	0.749
Global RVD, %	11 [8–17]	12 [8–18]	0.336	11 [7–16]	0.936
RVD _non−dep_, %	14 [11–20]	14 [9–24]	0.336	13 [9–21]	0.749
RVD _dep_, %	9 [6–13]	9 [7–13]	0.654	9 [6–11]	0.936

Variables are expressed as median, with an interquartile range [IQR]. *p-value a-c: **0.018** and **0.006**, respectively. a = Standard HFNC; b = Asymmetrical HFNC; c = Venturi mask

*Abbreviations*: *HFNC* high-flow nasal cannula, *DE* diaphragmatic excursion, *TFdi* diaphragmatic thickening fraction, *VT* tidal volume, *dep* dependent, *non-dep* non-dependent, *EELI* delta end-expiratory lung impedance (measured as percent change from Venturi mask), *GI* global inhomogeneity index, *RVD* regional ventilation delay, *MV* minute ventilation, *VM* Venturi mask, *N* number

### Gas exchange, dyspnea, and comfort

As shown in Table 3, respiratory rate, SaO_2_, PaO_2_/set FiO_2_, gas exchange and hemodynamic parameters were not different between asymmetrical and standard HFNC. Comfort, but not dyspnea, was higher during asymmetrical HFNC, compared to the conventional interface (p = 0.044 and p = 0.763, respectively) (Table 3).

**Table 3:** Gas exchange, hemodynamic parameters, dyspnea, and comfort

Variable	Standard HFNC^a^ (N = 20)	Asymmetrical HFNC^b^ (N = 20)	Adjusted p-value (a-b)	Venturi mask^c^ (n = 20)	Adjusted p-value (b-c)
Set FiO_2_, %	40 [40–40]	40 [38–40]	0.763	40 [40–40]	0.104
Respiratory rate*min^− 1^	14 [10–16]	14 [11–18]	0.173	18 [13–19]	**0.007**
pH	7.46 [7.43–7.48]	7.46 [7.42–7.50]	0.763	7.43 [7.41–7.46]	0.350
SaO_2_, %	97 [96–98]	97 [97–98]	0.763	96 [94–97]	**0.006**
PaO_2_/set FiO_2_, mmHg	237 [211–322]	247 [225–300]	0.173	195 [177–259]	**0.001**
PaCO_2_, mmHg	42 [39–44]	42 [39–45]	0.763	41 [39–43]	0.492
SBP, mmHg	135 [122–152]	138 [122–158]	0.763	135 [126–164]	0.236
DBP, mmHg	63 [55–72]	64 [56–71]	0.763	65 [59–72]	0.452
Comfort (range 0–10)	8 [7–9]	10 [7–10]	**0.044**	8 [6–10]	0.104
Dyspnea (range 0–10)	0 [0–1]	0 [0–1]	0.763	0 [0–2]	0.104

Variables are expressed as median, with an interquartile range [IQR]. a = Standard HFNC; b = Asymmetrical HFNC; c = Venturi mask

*Abbreviations*: *HFNC* high-flow nasal cannula, *PaO*_*2*_ arterial partial pressure of oxygen, *PaCO*_*2*_ arterial partial pressure of carbon dioxide, *FiO*_*2*_ inspiratory fraction of oxygen, *SBP* systolic blood pressure, *DBP* diastolic blood pressure, *SaO*_*2*_ arterial oxygen saturation, *N* number

### Asymmetrical HFNC vs. venturi mask

Asymmetrical HFNC oxygen therapy decreased corrected MV and respiratory rate, as compared to VM (p = 0.050 and p = 0.007, respectively); while SaO_2_ and PaO_2_/set FiO_2_ were higher during asymmetrical HFNC, as compared to VM (p = 0.006 and p = 0.001, respectively) (Table 2). Finally, both comfort and dyspnea were not different as compared to VM (p = 0.104) (Table 3). Additional data on asymmetrical HFNC oxygen therapy, as compared to VM, are reported in the Supplementary materials (Table S3).

### Subset analysis

Additional analyses were performed considering only patients (14, 70%) improving lung aeration using standard HFNCs, as shown in the Supplementary materials (Tables S4 and S5). Once again, the asymmetrical interface, as compared to the standard configuration, shows higher patient comfort (p = 0.016).

## Discussion

In this pilot physiological study, randomizing 20 ICU patients with post-extubation hARF, the use of the asymmetrical HFNC, despite showing similar performances in terms of lung aeration, TFdi, DE, ventilatory efficiency, and gas exchange, was associated with improved patient comfort, compared to standard HFNC interface.

While conventional HFNC has been shown to generate a ‘PEEP effect’, promoting alveolar recruitment and improving oxygenation, with a flow-dependent increase in global EELI, a valid surrogate of alveolar recruitment, clinical evidences on the potential benefits of the use of asymmetrical prongs on lung aeration are still lacking [2, 5–8, 18].

Recent bench studies, collecting data from anatomically ‘correct’ three-dimensional upper airway models, showed that an increase in asymmetrical nare occlusion led to a significant improvement of the ‘PEEP effect’ [17, 37, 38]. On the contrary, in 10 ICU patients affected by mild-to-moderate hARF, Slobod et al. showed that the asymmetrical HFNC interface did not affect alveolar recruitment, dorsal fraction of ventilation and end-expiratory lung impedance, thus suggesting no major effect on alveolar aeration [18]. Our results are in keeping with those findings despite uneven populations. Indeed, in our cohort of adults experiencing post-extubation hARF, EELI was similar between asymmetrical and standard configuration, without any difference between ventral and dorsal aeration. However, our results on lung aeration may be limited because, first, we did not measure EELV directly with computed tomography [39], but only through a derived EIT parameter (i.e., EELI) and, second, because we cannot exclude that some patients breathe with their mouths open, which may decrease the ‘PEEP effect’ associated with HFNC oxygen therapy [11, 40]. However, all above mentioned bench studies, describing an increased ‘PEEP effect’ during the asymmetrical HFNC, suffer an important limitation worthy of discussion, such as collecting data from ‘normal’ upper airway models, and not accounting for anatomical abnormalities that may affect nasal flow distribution and ‘PEEP effect’ [17, 37, 38].

Likewise, data on the role of asymmetrical nostrils in reducing the patients’ work of breathing are still conflicting. Interestingly, Slobod et al. found that the inspiratory esophageal pressure-time product was slightly reduced with the asymmetrical HFNC, in comparison with the standard interface [18]. Since we cannot exclude that the presence of the esophageal catheter, useful for measuring the esophageal pressure-time product or the diaphragm electrical activity, may affect either the dead space clearance or the ‘PEEP effect’, we decided to remove any nasogastric tube before protocol initiation [37, 41]. As an alternative, we decided to explore the patient inspiratory effort by ultrasound assessment, a less invasive technique, with easy learning and high reproducibility [11, 40, 42]. Based on our findings, both TFdi and DE were similar between different interfaces.

Furthermore, standard HFNC support has been shown to reduce dead space and to improve CO_2_-washout in mixed populations (i.e., hypoxemic ICU patients, hypercapnic COPD subjects ect) [1]. In keeping with those previous findings, Tatkov et al. showed an increased CO_2_-washout, and Slobod et al. observed an increased ventilatory efficiency during asymmetrical HFNC support, as compared to the standard interface [17, 18]. According to our findings, the asymmetrical HFNC performed similarly to the standard interface, probably due to an important heterogeneity in patients’ baseline characteristics that may affect the comparison with the above-mentioned study [18]. Indeed, in our cohort only 4 out of 20 (20%) patients were intubated for ‘primary’ ARF or pneumonia (i.e., patient n. 1, 3, 4, 17), while Slobod et al. enrolled 6 out of 10 (60%) patients with ‘primary’ ARF [18].

Finally, despite the absence of relevant differences between the standard and asymmetrical interface, our results suggest greater comfort during the asymmetrical HFNC interface, favoring their application routinely. The reasons why our patients reported greater comfort during the asymmetrical interface are not entirely clear. However, our results seem to be promising for realizing further studies investigating the impact of asymmetrical cannulas on lung aeration and diaphragm activity in different clinical settings, with different patient selection.

Our study has some limitations. First, during our trial, it was not possible to control the potential impact of spontaneous patient movements on EIT recordings [26], although we marked the initial EIT belt position, as previously described [26]. Second, we cannot exclude that the absence of a ‘PEEP effect’ may be due to the potential impact of mouth breathing during HFNC, as previously described [11, 40]. Third, we enrolled patients with a median invasive mechanical ventilation of 30 h. So doing, the effect on alveolar recruitment and CO_2_ clearance in case of longer endotracheal intubation remains unclear and further studies are required to clarify this issue. Finally, due to the explorative nature of our investigation we cannot exclude that our study could be underpowered to assess any difference between the asymmetrical and standard interface. In fact, our sample size could not be enough to measure a possible effect on lung aeration and diaphragm activity. In addition, further studies are necessary to explore the impact of asymmetrical HFNCs in different clinical settings, with different patient selection.

In conclusion, in acute post-extubation hARF, the use of the asymmetrical HFNC, as compared to standard HFNC, improved patient comfort slightly, despite similar performances in terms of lung aeration, TFdi, DE, ventilatory efficiency, dyspnea and gas exchange.

### Electronic supplementary material

Below is the link to the electronic supplementary material.

Supplementary Material 1: Clinical trial protocol (last released version)

Supplementary Material 2: Additional data on diaphragm ultrasound evaluation, EIT and gas exchanges

## Data Availability

The datasets used and/or analysed during the current study are available for the corresponding author on reasonable request.

## Abbreviations

BMI: body mass index
CO_2_: carbon dioxide
COPD: chronic obstructive pulmonary disease
DBP: diastolic blood pressure
DE: diaphragm excursion
dep: dependent
EELI: end-expiratory lung impedance
EIT: electrical impedance tomography
EELV: end-expiratory lung volume
GI: global inhomogeneity index
HFNC: high-flow nasal cannula
hARF: hypoxemic acute respiratory failure
ICU: intensive care unit
IMV: invasive mechanical ventilation
IQR: interquartile range
LOS: length of stay
MV: minute ventilation
N: number
non-dep: non-dependent
NRS: numerical scale
PaO_2_: arterial partial pressure of oxygen
PaCO_2_: arterial pressure of carbon dioxide
PEEP: positive end-expiratory pressure
RVD: regional ventilation delay
RR: respiratory rate
SAPS II: simplified acute physiology score II PaO_2_
SaO_2_: arterial oxygen saturation
SBP: systolic blood pressure
Tot: total
V_T_: tidal volume
TFdi: diaphragm ultrasound thickening fraction
VM: Venturi mask
