# Supplementary material for: Asymmetrical high-flow nasal cannula performs similarly to standard interface in patients with acute hypoxemic post-extubation respiratory failure: a pilot study
Source: BMC Pulm Med. 2024 Jan 8;24:21. doi: 10.1186/s12890-023-02820-x (PMC10775427; doi:10.1186/s12890-023-02820-x)
Supplement: Supplementary file 1 — Supplementary Material 1: Clinical trial protocol (last released version) [file 12890_2023_2820_MOESM1_ESM.pdf]

ClinicalTrials.gov PRS DRAFT Receipt (Working Version)
Last Update: 12/18/2023 08:37

ClinicalTrials.gov ID: NCT05838326

Study Identification

Unique Protocol ID: AOP2949

Brief Title: 'Optiflow + Duet' Interface' vs 'Standard' High Flow Nasal Cannula (OPTIMARF)

Official Title: Physiological Effects of High Flow Oxygen Therapy Using 'Optiflow + Duet' Interface vs 'Standard' High Flow Nasal Cannula for Acute Respiratory Failure After Extubation. The OPTIMARF Study.

Secondary IDs:

Study Status

Record Verification: December 2023

Overall Status: Completed

Study Start: May 8, 2023 [Actual]

Primary Completion: June 30, 2023 [Actual]

Study Completion: July 30, 2023 [Actual]

Sponsor/Collaborators

Sponsor: University of Padova

Responsible Party: Sponsor

Collaborators:

Oversight

U.S. FDA-regulated Drug: No

U.S. FDA-regulated Device: No

Unapproved/Uncleared Device: No

U.S. FDA IND/IDE: No

Human Subjects Review: Board Status: Approved
Approval Number: AOP2949
Board Name: Ethical Commitee of Padua University Hospital
Board Affiliation: Azienda Ospedale-Università Padova
Phone: 049-821
Email: ce.sperimentazione@aopd.veneto.it
Address:

2, Giustiniani street, 35120 Padova, Italy

Data Monitoring:	Yes
FDA Regulated Intervention:	No

Study Description

Brief Summary: High-flow nasal cannula (HFNC) therapy is increasingly used in the management of acute respiratory failure. Its clinical application has been largely investigated in chronic obstructive pulmonary disease (COPD) patients but only marginally in patients experiencing acute respiratory failure after extubation. Promising data have been published in vitro about new asymmetrical high flow nasal cannula, named 'Optiflow + DUET'. Positive airway pressure, that dynamically changes with breathing, and clearance of anatomical dead space are the key mechanisms of noninvasive respiratory support with nasal high flow. Pressure mainly depends on flow rate and nare occlusion. The hypothesis is that an increase in asymmetrical occlusion of the nares leads to an improvement in dead-space clearance resulting in a reduction in re-breathing and breathing work.

Detailed Description: A prospective cross-over RCT on the effects of 'Optiflow + DUET' as compared to convetional symmetrical high flow cannula and Venturi mask.

Conditions

Conditions: Hypoxemia

Keywords: asymmetrical nasal cannula
hypoxemia
DUET

Study Design

Study Type:	Interventional
Primary Purpose:	Supportive Care
Study Phase:	N/A
Interventional Study Model:	Crossover Assignment
Number of Arms:	2
Masking:	None (Open Label)
Allocation:	Randomized
Enrollment:	20 [Actual]

Arms and Interventions

Arms	Assigned Interventions
Active Comparator: Symmetrical high flow nasal cannula (HFNO)<br>After a 'baseline' trial using Venturi Mask, within the first 120 minutes after extubation, and assessing a arterial oxygen pressure (PaO2) and inspiratory oxygen fraction (FiO2) ratio < 300, patients will be randomly assigned to a first 1h-phase of 'conventional HFNO' or 'DUET HFNO'. At the end of each session	Device: Symmetrical high flow nasal cannula (HFNO)<br>After a 'baseline' trial using Venturi Mask, within the first 120 minutes after extubation, and assessing a PaO2/FiO2 ratio < 300, patients will be randomly assigned to a first 1h-phase of 'conventional HFNO' or 'DUET HFNO'. At the end of each session several clinical parameters (i.e. DUS, EIT, ABGs, comfort, VAS) will be collected. Specifically, Gas-flow rate will be set at a maximum of 60 L/min, temperature at

Arms	Assigned Interventions
several clinical parameters (i.e. DUS, EIT, ABGs, comfort, VAS) will be collected.<br><br>Specifically, Gas-flow rate will be set at a maximum of 60 L/min, temperature at a maximum of 37°C, while FiO2 will be adjusted to maintain a peripheral saturation (SpO2) between 92 and 98%.	a maximum of 37°C, while FiO2 will be adjusted to maintain SpO2 between 92 and 98%.
Active Comparator: Asymmetrical high flow nasal cannula (DUET HFNO)<br>After a 'baseline' trial using Venturi Mask, within the first 120 minutes after extubation, and assessing a PaO2/FiO2 ratio < 300 (as described above), patients will be randomly assigned to a second 1h-phase of 'DUET HFNO' vs 'conventional HFNO'. At the end of each session several clinical parameters (i.e. DUS, EIT, ABGs, comfort, VAS) will be collected.<br><br>Specifically, Gas-flow rate will be set at a maximum of 60L/min, temperature at a maximum of 37°C, while FiO2 will be adjusted to maintain SpO2 between 92 and 98%.	Device: Asymmetrical high flow nasal cannula (DUET HFNO)<br>After a 'baseline' trial using Venturi Mask, within the first 120 minutes after extubation, and assessing a PaO2/FiO2 ratio < 300, patients will be randomly assigned to a first 1h-phase of 'DUET HFNO' vs 'conventional HFNO'. At the end of each session several clinical parameters (i.e. DUS, EIT, ABGs, comfort, VAS) will be collected. Specifically, Gas-flow rate will be set at a maximum of 60 L/min, temperature at a maximum of 37°C, while FiO2 will be adjusted to maintain SpO2 between 92 and 98%. A 5-10min 'washout' phase using VM, between different interfaces, will be allowed.

Outcome Measures

Primary Outcome Measure:

1. Breathing effort
   To evaluate changes, between arms, in breathing effort (as assessed by diaphragm ultrasound (DUS)).

   [Time Frame: Last 10 minutes of 1 hour-trial]
2. Lung aeration
   To evaluate changes, between arms, in lung aeration (as assessed by the end-expiratory lung impedance (delta EELI) through EIT.

   [Time Frame: Last 10 minutes of 1 hour-trial]

Secondary Outcome Measure:

3. Minute ventilation (MV) (L/min)
   To evaluate breathing pattern

   [Time Frame: Last 10 minutes of 1 hour-trial]
4. corrected MV (L/min)
   To evaluate breathing pattern

   [Time Frame: Last 10 minutes of 1 hour-trial]
5. Breathing heterogeneity
   To evaluate changes, between arms, in ventilation distribution (as assessed by pendelluft (yes/not), respiratory rates/min, tidal volume (ml), global inhomogeneity index through EIT)

   [Time Frame: Last 10 minutes of 1 hour-trial]
6. Comfort
   To evaluate changes in comfort, between arms, as assessed by NRS scale

   [Time Frame: Last 10 minutes of 1h-trial]
7. Dyspnea
   To evaluate changes in dyspnea, between arms, as assessed by VAS scale

   [Time Frame: Last 10 minutes of 1h-trial]

8. Gas exchange
   To evaluate changes, between arms, in gas exchange (as assessed by PaO2 mmHg, pCO2 mmHg, pH through ABGs)

   [Time Frame: Last 10 minutes of 1h-trial]

Eligibility

Minimum Age: 18 Years

Maximum Age:

Sex: All

Gender Based: No

Accepts Healthy Volunteers: No

Criteria: Inclusion Criteria:

- age >18 years old;
- invasive mechanical ventilation > 24h;
- acute respiratory failure within 120 minutes after extubation (defined during a spontaneous trial using Venturi mask and assessing a PaO2/FiO2 ratio < 300;
- absence of Sars-Cov-2 positivity;
- absence of cardiological or long-term respiratory disease

Exclusion Criteria:

- pregnancy
- tracheostomy
- non-invasive ventilation after extubation
- second tracheal intubation
- contraindications for EIT belt
- facial or nose abnormalities

Contacts/Locations

Central Contact Person: Annalisa Boscolo, MD
Telephone: +393498324972
Email: annalisa.boscolobozza@unipd.it

Central Contact Backup:

Study Officials: Annalisa Boscolo, MD
Study Principal Investigator
AOUP, DIMED

Locations: **Italy**
Institute of Anaesthesia and Intensive Care, Padua University hospital
Padova, Italy, 35120
Contact: Annalisa Boscolo, MD, PhD +393498324972
annalisa.boscolobozza@aopd.veneto.it

IPDSharing

Plan to Share IPD:

References

Citations:

Links:

Available IPD/Information:

U.S. National Library of Medicine | U.S. National Institutes of Health | U.S. Department of Health & Human Services
