# Supplementary material for: Asymmetrical high-flow nasal cannula performs similarly to standard interface in patients with acute hypoxemic post-extubation respiratory failure: a pilot study
Source: BMC Pulm Med. 2024 Jan 8;24:21. doi: 10.1186/s12890-023-02820-x (PMC10775427; doi:10.1186/s12890-023-02820-x)
Supplement: Supplementary file 2 — Supplementary Material 2: Additional data on diaphragm ultrasound evaluation, EIT and gas exchanges [file 12890_2023_2820_MOESM2_ESM.docx]

**SUPPLEMENTAL MATERIAL**

**Title: *'Asymmetrical high-flow nasal cannula performs similarly to standard interface in patients with acute hypoxemic post-extubation respiratory failure:a pilot study'***

**Figure S1. Additional data on electrical impedance tomography during standard and asymmetrical HFNC oxygen therapy**, page 2

**Figure S2. Additional data on diaphragm ultrasound evaluation during standard and asymmetrical HFNC oxygen therapy**, page 3

**Table S~~1~~3. Additional data on electrical impedance tomography parameters and diaphragm measurements (asymmetrical HFNC vs. Venturi mask),** page ~~2~~4

**Table S4. Subset analysis: electrical impedance tomography parameters and diaphragm measurements**, page 5

**Table S5. Subset analysis: gas exchange, hemodynamic parameters, dyspnea, and comfort**, page 6

**Figure S1. Additional data on electrical impedance tomography during standard and asymmetrical HFNC oxygen therapy**.

**
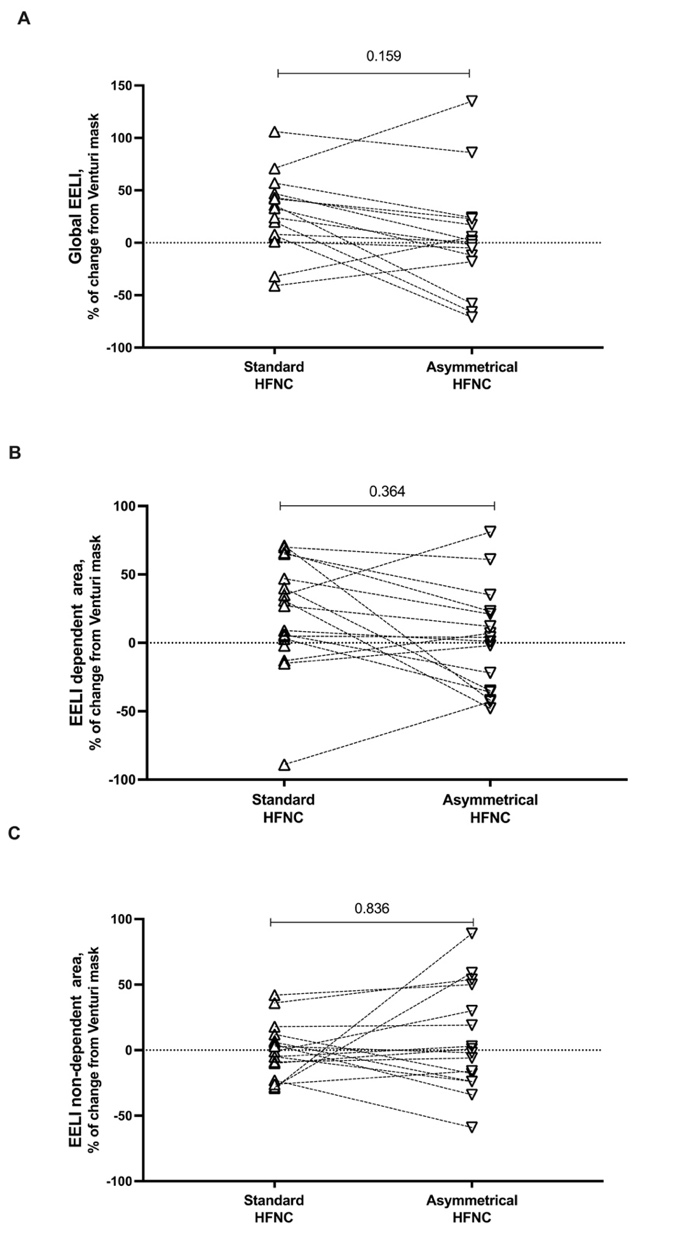
**

Variables are expressed as absolute numbers. **A**: global lung aeration; **B**: lung aeration in dependent area; **C**: lung aeration in non-dependent area. *Abbreviations*: ns = not significant; HFNC = high-flow nasal cannula; dep = dependent; non-dep = non-dependent; EELI = end-expiratory lung impedance (measured as percent change from VM).

**Figure S2. Additional data on diaphragm ultrasound evaluation during standard and asymmetrical HFNC oxygen therapy**.


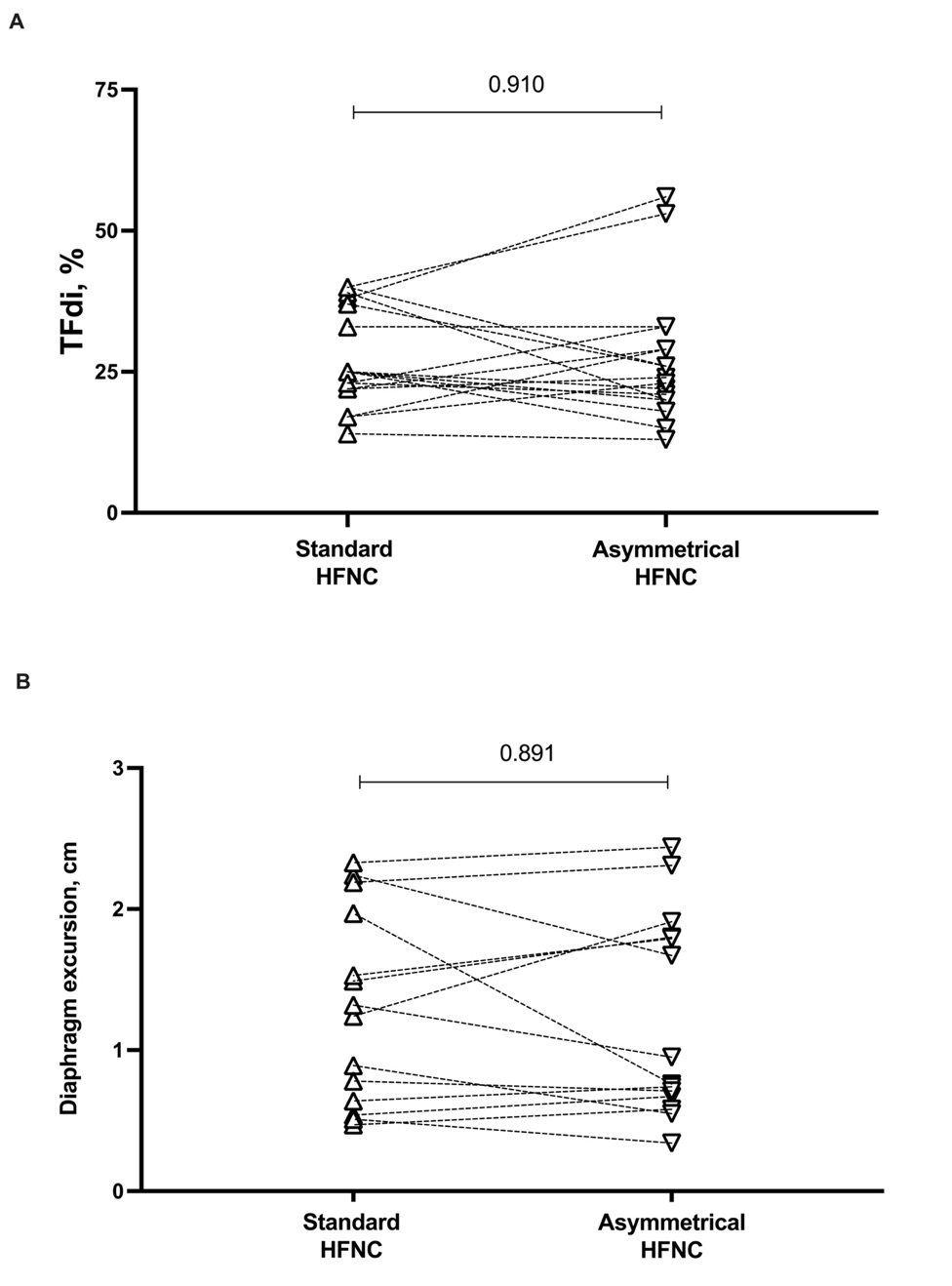


Variables are expressed as absolute numbers. **A**: TFdi; **B**: diaphragm excursion; *Abbreviations*: HFNC = high-flow nasal cannula; TFdi = diaphragm thickening fraction.

**Table S3. Additional electrical impedance tomography parameters and diaphragm measurements (asymmetrical HFNC vs. Venturi mask).**

| **Variable** | **Venturi mask**  **(N=20)** | **Asymmetrical HFNC**  **(N=20)** | **Adjusted**  **p-value** |
| --- | --- | --- | --- |
|  |  |  |  |
| Global EELI  (change from VM), % | - | -0.5 [-17, 22] | 0.991* |
| EELI _non-dep_  (change from VM), % | - | -0.5 [-23, 45] | 0.991* |
| EELI _dep_  (change from VM), % | - | 2.5 [-36, 23] | 0.991* |
|  |  |  |  |
| TFdi, % | 28 [18-41] | 24 [20-30] | 0.991* |
| DE, cm | 1.54 [0.81-2.21] | 0.86 [0.65-1.83] | 0.740* |
|  |  |  |  |

Variables are expressed as median, with an interquartile range [IQR]. *p-values between Venturi mask and standard HFNC: **0.006,** 0.845, **0.031**, 0.211, 0.142**,** respectively. *Abbreviations*: HFNC = high-flow nasal cannula; DE = diaphragmatic excursion; TFdi = diaphragmatic thickening fraction; EELI = end-expiratory lung impedance (measured as percent change from Venturi mask); VM = Venturi mask; N = number.

**Table S4. Subset analysis: electrical impedance tomography parameters and diaphragm measurements.**

| **Variable** | **Standard HFNC^a^**  **(N=14)** | **Asymmetrical HFNC^b^**  **(N=14)** | **p-value**  **(a-b)** | **Venturi mask^c^**  **(n=14)** | **p-value**  **(b-c)** |  |
| --- | --- | --- | --- | --- | --- | --- |
|  |  |  |  |  |  |  |
| Global EELI  (change from VM), % | 35 [8-50] | 1 [-35, 24] | **0.001** | - | 0.800* |  |
| EELI _non-dep_  (change from VM), % | 4 [-9-14] | -2 [-24, 55] | 0.624 | - | 0.905 |  |
| EELI _dep_  (change from VM), % | 33 [6-65] | 4 [-36, 29] | **0.011** | - | 0.853 |  |
|  | |  |  |  |  |  |
| TFdi, % | 25 [22-38] | 26 [22-33] | 0.713 | 30 [21-45] | 0.807* |  |
| DE, cm | 1.49 [0.89-2.19] | 1.67 [0.71-1.91] | 0.577 | 2 [1.44-2.31] | 0.250* |  |
|  | |  |  |  |  |  |
| MV (change from VM), % | -13 [-27, 5] | -15 [-20, 5] | 0.594 | - | 0.092* |  |
| Corrected MV  (change from VM), % | -9 [-22, 4] | -11 [-19, 1.34] | 0.893 | - | 0.068* |  |

Only patients improving global EELI during standard HFNC were analyzed. Variables are expressed as median, with an interquartile range [IQR]. *p-value a-c: **<0.001**, 0.328, 0.359, 0.080, 0.068, respectively. a=Standard HFNC; b=Asymmetrical HFNC; c=Venturi mask. *Abbreviations*: HFNC = high-flow nasal cannula; DE = diaphragmatic excursion; TFdi = diaphragmatic thickening fraction; dep = dependent; non-dep = non-dependent; EELI = delta end-expiratory lung impedance (measured as percent change from Venturi mask); MV = minute ventilation; VM = Venturi mask; N = number.

**Table S5. Subset analysis: gas exchange, dyspnea, and comfort.**

| **Variable** | **Standard HFNC^a^**  **(N=14)** | **Asymmetrical HFNC^b^**  **(N=14)** | **p-value**  **(a-b)** | **Venturi mask^c^**  **(n=14)** | **p-value**  **(b-c)** |
| --- | --- | --- | --- | --- | --- |
| pH | 7.45 [7.42-7.49] | 7.44 [7.42-7.50] | 0.689 | 7.45 [7.41-7.50] | 0.641* |
| PaO_2_/set FiO_2_, mmHg | 237 [209-286] | 247 [224-296] | 0.502 | 191 [174-237] | **<0.001*** |
| PaCO_2_, mmHg | 41 [38-44] | 41 [39-45] | 0.659 | 41 [39-44] | 0.417* |
| Comfort (range 0-10) | 8 [6-9] | 10 [7-10] | **0.016** | 8 [6-10] | 0.094* |
| Dyspnea (range 0-10) | 0 [0-2] | 0 [0-2] | 0.750 | 0 [0-2] | 0.063* |

Only patients improving global EELI during standard HFNC were analysed. Variables are expressed as median, with an interquartile range [IQR]. *p-value a-c: 0.205, **<0.001**, 0.999, 0.910, 0.563, respectively. a=Standard HFNC; b=Asymmetrical HFNC; c=Venturi mask. *Abbreviations*: HFNC = high-flow nasal cannula; PaO_2_ = arterial partial pressure of oxygen; PaCO_2_ = arterial partial pressure of carbon dioxide; FiO_2_ = inspiratory fraction of oxygen; N = number.
